# Clinical Practice and Diagnostic Trends in Hereditary Transthyretin Amyloidosis: A 25-Year Observational Study

**DOI:** 10.3390/medicina62050907

**Published:** 2026-05-07

**Authors:** Kaori Sumi, Teruaki Masuda, Hidekazu Kondo, Shotaro Saito, Yasuhiro Aso, Yusuke Hazama, Konen Obayashi, Toshiya Nomura, Mitsuharu Ueda, Naohiko Takahashi, Noriyuki Kimura

**Affiliations:** 1 Department of Neurology, Faculty of Medicine, Oita University, Oita 879-5593, Japan; ka0941043@oita-u.ac.jp (K.S.); noriyuki@oita-u.ac.jp (N.K.); 2 Department of Cardiology and Clinical Examination, Faculty of Medicine, Oita University, Oita 879-5593, Japan; hkondo@oita-u.ac.jp (H.K.); sait1982@oita-u.ac.jp (S.S.); takanao@oita-u.ac.jp (N.T.); 3 Department of Neurology, Oita Prefectural Hospital, Oita 870-851, Japan; yasuhiroaso@oita-u.ac.jp; 4 Department of Neurology, Shin Beppu Hospital, Oita 874-8538, Japan; yhazddt@gmail.com; 5 Department of Clinical Physiology, Graduate School of Life Sciences, Kumamoto University, Kumamoto 862-0976, Japan; konen@kumamoto-u.ac.jp; 6 Department of Neurology, Graduate School of Medical Sciences, Kumamoto University, Kumamoto 860-8556, Japan; caramelbox0106@gmail.com (T.N.); mitt@rb3.so-net.ne.jp (M.U.)

**Keywords:** hereditary transthyretin amyloidosis, transthyretin, non-endemic areas, disease-modifying drugs

## Abstract

*Background and Objectives*: Hereditary transthyretin amyloidosis (ATTRv), a multisystemic disorder caused by transthyretin (TTR) gene mutations, exhibits phenotypic heterogeneity that can hamper recognition in non-endemic areas. Here, we investigated the clinical practice of ATTRv amyloidosis over an extended period and examined the challenges currently faced in non-endemic regions. *Materials and Methods*: We conducted an observational study of 18 patients with ATTRv amyloidosis diagnosed at Oita University and its affiliated hospitals between 2000 and 2025, using both retrospective and prospective data collection, to evaluate clinical features, treatments, and outcomes. *Results*: The median age at disease onset was 64 years, and 27.8% of patients had a family history of the disease. Val30Met (V30M) was the most common (55.6%) mutation; Tyr114Ser was the most common non-V30M variant. Sensory disturbances (50%) were the most common initial symptoms, followed by cardiac symptoms (38.9%). The cardiology department most frequently diagnosed ATTRv amyloidosis, followed by the neurology department. Two patients were relatives of previously diagnosed probands and were therefore identified during their first visit to the initial department. New diagnoses increased over time (1 in 2000–2009, 7 in 2010–2019, and 10 in 2020–2025), although diagnostic delays persisted. After the therapeutic agents shifted from patisiran to vutrisiran, the serum TTR value decreased (*p* = 0.0078) without significant deterioration in cardiac parameters. In the current treatment era, longer-term survivors have been observed, but multiple organ dysfunction has become more apparent, and ocular manifestations have emerged as a clinically important problem, particularly in patients with longer disease duration (*p* = 0.0169). *Conclusions:* Physicians in various departments must remain vigilant for the presence of ATTRv amyloidosis. Addressing challenges faced by long-term survivors, including ocular manifestations and central nervous system complications, has become crucial, even in non-endemic areas.

## 1. Introduction

Hereditary transthyretin amyloidosis (ATTRv) is an autosomal-dominant, progressive systemic disorder caused by mutations in the transthyretin (TTR) gene [1,2]. This disease produces a wide range of clinical manifestations, including sensorimotor polyneuropathy, autonomic dysfunction, cardiac disorders, ocular involvement, and central nervous system manifestations [3]. To date, more than 150 TTR gene mutations have been identified. The clinical heterogeneity associated with phenotypic and genotypic variations in ATTRv amyloidosis complicates early diagnosis, particularly in non-endemic areas, and may lead to misdiagnosis [4,5,6].

Innovative disease-modifying drugs (DMDs) for ATTRv amyloidosis, which include TTR tetramer stabilizers, TTR gene-silencing therapies that use small interfering ribonucleic acid, and antisense oligonucleotides, have recently been approved in several countries [7,8,9,10,11]. These DMDs have significantly improved the prognosis and quality of life for patients with the early-onset Val30Met (p.Val50Met; V30M) mutation in endemic areas, as well as for patients with late-onset V30M and those with non-V30M mutations in non-endemic areas, where conventional treatments such as liver transplantation are challenging to apply [12,13]. Clinical challenges faced by long-term survivors are therefore becoming increasingly obvious in non-endemic areas.

In this study, we investigated the clinical practice of ATTRv amyloidosis management during an extended period and analyzed the related challenges currently encountered in our region.

## 2. Materials and Methods

### 2.1. Study Population

We conducted an observational study of 18 consecutive patients diagnosed with ATTRv amyloidosis at Oita University and its main affiliated hospitals (Oita Prefectural Hospital and Shin Beppu Hospital) between 2000 and 2025, using both retrospective and prospective data collection. All patients were followed until December 2025. A definitive diagnosis of ATTRv amyloidosis was established based on the identification of a pathogenic *TTR* variant by genetic testing, clinically compatible findings, and histopathological evidence of TTR amyloid deposition [14,15]. AL amyloidosis was ruled out based on serum and urine immunofixation electrophoresis, free light-chain assays, and histopathological findings. The study protocol was approved by the Oita University Research Ethics Committee (Nos. 2329 and 2640), and all participants gave their informed consent for their involvement in the study.

### 2.2. Clinical Examinations

We evaluated the clinical features of patients with ATTRv amyloidosis, including basic information, TTR gene variants, origin in endemic or non-endemic areas, family history, initial symptoms, clinical manifestations, laboratory findings, treatment, and outcomes of treatment, and analyzed factors associated with ATTRv amyloidosis [14,16]. We also analyzed the diagnostic process for ATTRv amyloidosis during three distinct time periods: (i) 2000–2009, (ii) 2010–2019, and (iii) 2020–2025. In addition, we performed a comprehensive assessment of values for serum TTR, N-terminal pro-brain natriuretic peptide (NT-proBNP), and high-sensitivity troponin T, as well as echocardiographic assessments of patients who switched from patisiran therapy to vutrisiran therapy at the start of treatment and after 1 year. We also collected data on the patients’ treatment histories with DMDs.

### 2.3. Statistical Analysis

Statistical analyses included the Mann–Whitney U-test, Wilcoxon signed-rank test for data analysis, and Fisher’s exact test. We used GraphPad Prism 7.05 for Windows (GraphPad Software, San Diego, CA, USA) to perform all analyses. We selected *p* values of less than 0.05 as statistically significant.

## 3. Results

### 3.1. Clinical Characteristics of Patients with ATTRv Amyloidosis

Table 1 provides the clinical findings of 18 patients who had a definitive diagnosis of ATTRv amyloidosis. Five patients (27.8%) had a family history of ATTRv amyloidosis, with one patient having a father who carried the V30M mutation in an endemic area. Two familial pairs were identified: Cases 2 and 3 and Cases 12 and 13. Ten patients (55.6%) had the TTR variant V30M, whereas among the four non-V30M variants, the most common variant was Tyr114Ser (p.Tyr134Ser), which was found in four individuals (Table S1). The most frequent initial symptom was sensory disturbance, which occurred in nine (50%) patients. More cases of sensory disturbances developed in the upper limbs than in the lower limbs. In addition, seven patients (38.9%) presented with cardiac symptoms. Among the nine patients whose initial symptom was sensory disturbance, six initially visited the orthopedic department. No patient in the non-V30M group visited the neurology department at the initial visit. The departments that most frequently diagnosed ATTRv amyloidosis were cardiology (55.6%), followed by neurology (22.2%), orthopedics (16.7%), and ophthalmology (5.6%). Patients who had a diagnosis obtained by neurologists all had the V30M mutation, whereas those who had a diagnosis delivered by orthopedic surgeons had a non-V30M mutation. Cardiologists identified patients with different TTR variants. Two patients with a family history of ATTRv amyloidosis were diagnosed with the condition at the first department they visited. The detailed clinical characteristics of each patient are provided in Table 2.

### 3.2. Number of Patients Receiving New Diagnoses and Developing Initial Symptoms During Different Time Periods

**Figure 1:**
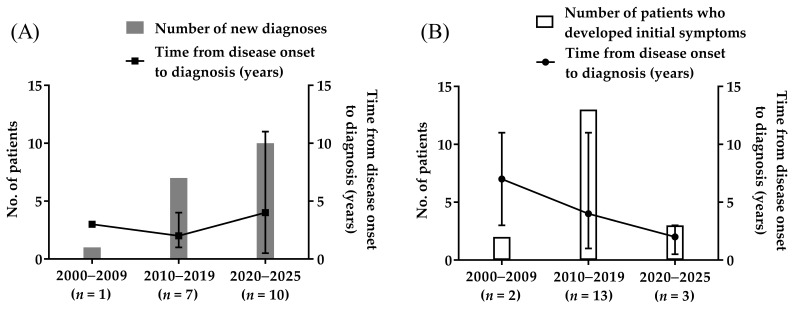
Twenty-five-year diagnostic trends in patients with ATTRv amyloidosis. This figure shows (**A**) the number of patients receiving new diagnoses and the times from disease onset to diagnosis, and (**B**) the number of patients who developed initial symptoms and the time from disease onset to diagnosis in each period. The points indicate median; error bars indicate range.

From 2000 to 2009, only one patient received a new diagnosis of ATTRv amyloidosis (Figure 1A, bar graph). This number increased to seven in the next decade (2010–2019). From 2020 to 2025, the number of patients with new diagnoses reached 10. The times from initial disease onset to diagnosis for these respective periods were 3.0 years, 2.0 years (range 1–4 years), and 4.0 years (range 0.5–11 years) (Figure 1A, line graph). The number of patients who developed initial symptoms of ATTRv amyloidosis was 2 from 2000 to 2009, 13 from 2010 to 2019, and 3 from 2020 to 2025 (Figure 1B, bar graph). The times from initial disease onset to diagnosis for these respective periods were 7.0 years (range 3–11 years), 4.2 years (range 1–11 years), and 1.8 years (range 0.5–3 years) (Figure 1B, line graph).

### 3.3. Treatment and Prognosis of Patients with ATTRv Amyloidosis

**Figure 2:**
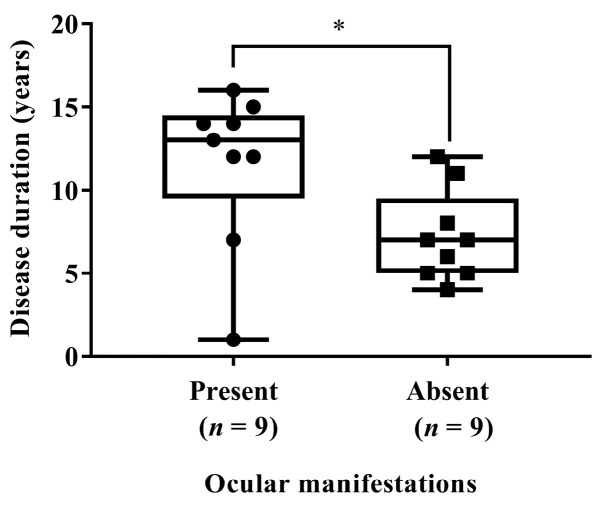
Disease duration from disease onset to death or final follow-up in patients with and without ocular manifestations of ATTRv amyloidosis. Patients with ocular manifestations had a significantly longer disease duration than those without ocular manifestations (Mann–Whitney U test, *p* = 0.0169). * *p* < 0.05.

Survivors had a median disease duration from ATTRv onset of 8.0 years (range 1–15 years); patients who died had a median disease duration from ATTRv onset to death of 13.0 years (range 5–16 years). Even when only one organ was initially affected, multiple organs were involved as the disease progressed (Table 3). Patients with ocular manifestations had a significantly longer disease duration, defined as the interval from symptom onset to death or final follow-up, than those without ocular manifestations (13 years [range, 1–16 years] vs. 7 years [range, 4–12 years]; *p* = 0.0169; Figure 2). Ocular symptoms were observed in 6/10 (60%) patients with V30M, 2/2 (100%) with Glu89Lys (p.Glu109Lys), and 1/1 (100%) with His56Arg (p.His76Arg), but in none of the four patients with Tyr114Ser. All ocular manifestations, including vitreous opacities, vitreous amyloid deposition, and glaucoma, were considered associated with ATTRv amyloidosis; no findings attributable to vitamin A deficiency were observed. Five patients died, with the causes of death being cachexia resulting from ATTRv amyloidosis (patient 1), heart failure due to cardiac amyloidosis (patients 2, 4, and 9), and cerebellar hemorrhage (patient 5). Patient 1 was not eligible for liver transplantation, which was the first-line treatment at the time, and thus received only symptomatic therapy. The remaining four patients were treated with tafamidis, patisiran, or vutrisiran. All surviving patients are now continuing to undergo treatment with vutrisiran. Detailed disease-modifying therapy histories for individual patients are provided in Supplementary Table S2. One year after switching from patisiran to vutrisiran, TTR levels were significantly lower than those after the final administration of patisiran (*n* = 8, *p* = 0.0078) (Supplemental Figure S1). In contrast, there were no statistically significant changes in levels of NT-proBNP (*n* = 8, *p* = 0.9453), troponin T (*n* = 7, *p* = 0.0938), left ventricle ejection fraction (*n* = 9, *p* > 0.9999), and interventricular septal thickness (*n* = 9, *p* = 0.4844), as well as the E/eʹ ratio (*n* = 9, *p* = 0.1328) in echocardiography.

## 4. Discussion

In this study, we provided a 25-year longitudinal view of the transition from the era of liver transplantation to the current era of multiple DMDs in a single non-endemic region. Although several clinical features observed in this study, including diagnostic delay, heterogeneous initial presentations, and the increasing role of cardiologists, have been reported previously, the unique contribution of this study is its long-term real-world perspective on ATTRv amyloidosis in a non-endemic setting. By capturing changes over time in clinical recognition, diagnostic approaches, therapeutic strategies, and outcomes, this study complements existing evidence from endemic cohorts, short-term observational studies, and clinical trials. Our study illustrates how diagnostic pathways have shifted over time, particularly with increased recognition by cardiologists, although diagnostic delays remain unresolved. In addition, this study highlights emerging long-term management issues, including ocular manifestations, multi-organ involvement, and possible central nervous system complications, which are becoming increasingly relevant in the era of disease-modifying therapies and prolonged survival.

First, our findings showed that cardiologists, as opposed to other specialists, most often diagnosed ATTRv amyloidosis in non-endemic areas, partly because cardiac symptoms were often the initial presentation in patients with V30M or non-V30M mutations [17,18]. Also, although sensory disturbances were the initial symptoms, these patients often received incorrect diagnoses, such as chronic inflammatory demyelinating polyneuropathy or lumbar spinal stenosis, until cardiac symptoms emerged later [19,20]. Another possible explanation has been the increasing number of cases in which TTR gene mutations were detected during the diagnostic process for suspected wild-type ATTR amyloidosis, which then led to a diagnosis of ATTRv amyloidosis [21,22]. As an interesting finding, at disease onset, sensory disturbances in the upper limbs were more frequent than those in the lower limbs. Patients with the V30M mutation in endemic areas typically had lower-limb sensory disturbances because of length-dependent peripheral neuropathy [4,23,24]. In contrast, patients in non-endemic areas often manifested carpal tunnel syndrome or upper-limb neuropathy [25,26]. Therefore, upper limb sensory disturbances are critical symptoms that physicians should not overlook when they evaluate ATTRv amyloidosis in these areas.

Although TTR amyloidosis remains rare, its clinical importance has increased in recent years because of advances in diagnostic modalities and DMDs. Consistent with this trend, the number of diagnoses of ATTRv amyloidosis has also increased worldwide [27,28]. Previously, liver transplantation was the primary therapy for ATTR amyloidosis, particularly for patients with early-onset V30M mutations in endemic areas, who were believed to be good candidates for this procedure [29,30]. However, a common issue was that numerous patients with late-onset V30M mutations in non-endemic areas or those with non-V30M mutations were not eligible for liver transplantation. The approval of DMDs, such as tafamidis, patisiran, and vutrisiran, which are expected to be effective for a wide range of patients in non-endemic regions, has increased awareness of this disease among healthcare providers in non-endemic regions [2]. A recent report indicated that the number of diagnoses increased more significantly in non-endemic areas than in endemic areas [17]. In the present study, we found that the number of new diagnoses was increasing, as was a trend toward earlier detection in recently diagnosed cases. However, some patients still experience delays in diagnosis. In patients who initially receive misdiagnoses, the initial diagnosis is often not reconsidered until additional organ impairment manifests. Although ATTRv amyloidosis is a hereditary disease, the absence of a family history does not exclude the diagnosis in non-endemic areas, because variable penetrance, late onset, small family size, or unrecognized disease in relatives may obscure the hereditary background [17,31,32]. Therefore, a timely and accurate diagnosis of patients in these areas remains an urgent challenge that requires improvement.

The reduction in serum TTR levels seen after switching from patisiran to vutrisiran in our cohort was consistent with findings from an observational crossover study [33]. However, this biochemical change was not accompanied by significant short-term improvements in cardiac biomarkers or echocardiographic parameters. One possible explanation is that a follow-up period of 1 year was too short to detect changes in cardiac function. In addition, because patients had already been treated with patisiran before the switch, their cardiac parameters may have been relatively stable at baseline, making any additional benefit of vutrisiran difficult to detect. Furthermore, comprehensive cardiac assessment, including cardiac MRI and pyrophosphate myocardial scintigraphy, was not performed in this study. Therefore, prospective studies with long-term follow-up and detailed cardiac assessment are needed to determine whether the greater TTR suppression achieved after switching from patisiran to vutrisiran has clinical significance.

The introduction of DMDs improved the survival outcomes of patients and increased the number of long-term survivors of ATTRv amyloidosis in our country [12]. Long-term survivors of ATTRv amyloidosis outside endemic regions therefore encountered long-term survivor-specific problems similar to those observed in endemic areas. Ocular manifestations such as glaucoma and vitreous opacities became more common over time. As with liver transplantation, severe ocular manifestations continued to reduce the quality of life, even after treatment with DMDs [34,35]. However, the presence or absence of ocular manifestations may reflect not only longer disease duration but also phenotypic differences among TTR variants. Although multiple organ dysfunction develops during the course of the disease, each manifestation has required symptomatic therapy, and multidisciplinary collaboration is essential for managing patients with ATTRv amyloidosis. Also, a patient with the V30M mutation died of severe cerebellar hemorrhage 14 years after disease onset. Because pathology and amyloid typing were not available, the etiology of the cerebellar hemorrhage could not be determined. Although ATTRv-related cerebral amyloid angiopathy may be considered as a possible mechanism, this remained unconfirmed. Importantly, currently approved DMDs do not adequately cross the blood-brain or blood–retinal barriers. TTR is produced locally by the choroid plexus and retinal pigment epithelium. Consequently, DMDs may not prevent local TTR amyloid deposition in these regions. For long-term survivors of ATTRv amyloidosis who have undergone DMD treatment, monitoring the development of ocular and central nervous system manifestations of ATTRv amyloidosis is critical [36,37].

This study had several limitations. First, the sample size was small because ATTRv amyloidosis is a rare disease, particularly in non-endemic areas. As a result, the statistical power was limited, and the results should be interpreted as exploratory observations rather than definitive evidence. The small sample size also limits the generalizability of our findings to the broader ATTRv population. Second, this study included a retrospective component, and information bias could not be completely excluded because initial symptoms, timing of onset, and diagnostic pathways were assessed using available medical records. Third, because this study was conducted at a university hospital and its affiliated hospitals, selection bias may have occurred. Patients referred to these institutions may have had more complex symptoms, more advanced disease, or a greater likelihood of undergoing detailed neurological, cardiac, ophthalmological, or genetic evaluations than the general ATTRv population in the region. Thus, our findings may not be fully generalizable to all patients with ATTRv amyloidosis in non-endemic areas. Fourth, some patients were from the same families, and familial clustering may have influenced the apparent frequency of specific variants and genotype–phenotype interpretations. Fifth, treatment-era effects, diagnostic awareness, and treatment availability changed substantially during the study period; therefore, this study was not designed to determine the prognostic effects of disease-modifying therapies. Finally, because *TTR* gene-silencing therapies have only recently become available, longer follow-up is needed to evaluate their effects on multiple organs. Prospective multicenter studies in diverse non-endemic areas are therefore warranted to confirm these findings.

## 5. Conclusions

In conclusion, this 25-year real-world cohort study in a non-endemic region showed that heterogeneous initial symptoms often prevent timely and accurate diagnosis of ATTRv amyloidosis. To ensure timely diagnosis and treatment, physicians in various departments must remain consistently vigilant for the presence of ATTRv amyloidosis during daily consultations. Also, for long-term management of ATTRv amyloidosis, physicians must carefully assess and provide treatment for conditions that are specific to long-term survivors of ATTRv amyloidosis.

## Figures and Tables

**Table 1:** Characteristics of patients at definitive diagnosis of ATTRv amyloidosis.

Characteristic	All Patients (*n* = 18)	Patients with V30M Mutation (*n* = 10)	Patients with Non-V30M Mutation (*n* = 8)	V30M vs. Non-V30M *p* Value
Age at disease onset, years, median [IQR]	64 [54.5–71]	63 [59–67]	66 [49–71]	0.7781
Sex, male/female	10/8	6/4	4/4	>0.9999
Family history, *n* (%)	5/18 (27.8)	1/10 (10)	4/8 (50)	0.1176
Time from disease onset to diagnosis, years, median [IQR]	3 [2–4]	3 [2–4]	3 [2–4.5]	0.8749
Initial symptoms				
Sensory disturbances, *n* (%)	9/18 (50)	5/10 (50)	4/8 (50)	>0.9999
Autonomic dysfunctions, *n* (%)	1/18 (5.6)	1/10 (10)	0/8 (0)	>0.9999
Cardiac symptoms, *n* (%)	7/18 (38.9)	3/10 (30)	4/8 (50)	0.6305
Ocular manifestations, *n* (%)	1/18 (5.6)	1/10 (10)	0/8 (0)	>0.9999
Department that the patient first visited				
Neurology, *n* (%)	3/18 (16.7)	3/10 (30)	0/8 (0)	0.2157
Cardiology, *n* (%)	8/18 (44.4)	4/10 (40)	4/8 (50)	>0.9999
Orthopedic surgery, *n* (%)	6/18 (33.3)	2/10 (20)	4/8 (50)	0.3213
Ophthalmology, *n* (%)	1/18 (5.6)	1/10 (10)	0/8 (0)	>0.9999
Department that provided the diagnosis				
Neurology, *n* (%)	4/18 (22.2)	4/10 (40)	0/8 (0)	0.0915
Cardiology, *n* (%)	10/18 (55.6)	5/10 (50)	5/8 (62.5)	0.6641
Orthopedic surgery, *n* (%)	3/18 (16.7)	0/10 (0)	3/8 (37.5)	0.0686
Ophthalmology, *n* (%)	1/18 (5.6)	1/10 (10)	0/8 (0)	>0.9999

Abbreviations: ATTRv, hereditary transthyretin amyloidosis; V30M, Val30Met. Data are either median [IQR] or number/total number (percentage). First-visited department refers to the department first consulted for the initial symptom.

**Table 2:** Baseline clinical characteristics and diagnostic pathways of individual patients with ATTRv amyloidosis.

Patient No.	Sex	Age at Disease Onset (Years)	Age at Diagnosis (Years)	TTR Variant	Area	Familial History	Initial Symptoms	Initial Diagnosis
1	F	59	62	Val30Met	N	(−)	Numbness in extremities	CTS, CIDP
2	F	49	50	Glu89Lys	N	Sister	Shortness of breath, edema	Cardiac amyloidosis
3	F	45	47	Glu89Lys	N	Sister	Numbness in both hands	Bilateral CTS
4	M	60	62	Tyr114Ser	N	(−)	Numbness in both hands	Bilateral CTS
5	M	57	60	Val30Met	N	(−)	Numbness in both legs	CIDP
6	F	65	67	Val30Met	N	(−)	Vitreous humor	Ocular amyloidosis
7	M	64	68	Val30Met	N	(−)	Palpitation	Paf
8	F	42	46	Val30Met	E	Father	Palpitation	ATTRv amyloidosis
9	M	61	72	Val30Met	N	(−)	Numbness in both hands	Heart failure, diabetic neuropathy
10	F	74	76	His56Arg	N	(−)	Shortness of breath	Cardiac amyloidosis
11	F	71	75	Thr49Ile	N	(−)	Tachycardia	HCM
12	M	71	75	Tyr114Ser	N	Brother	Numbness in both hands	ATTRv amyloidosis
13	M	68	73	Tyr114Ser	N	Brother	Numbness in both hands	Bilateral CTS
14	M	62	73	Val30Met	N	(−)	Dizziness, constipation	OH
15	M	73	75	Val30Met	N	(−)	Numbness in both hands	Drug-induced neuropathy
16	F	67	70	Val30Met	N	(−)	Numbness in both legs	LSS
17	M	80	80	Val30Met	N	(−)	Bradycardia	Cardiac amyloidosis
18	M	64	75	Tyr114Ser	N	(−)	Palpitation	Paf

Abbreviations: ATTRv, hereditary transthyretin amyloidosis; CIDP, chronic inflammatory demyelinating polyneuropathy; CTS, carpal tunnel syndrome; E, endemic area; F, female; HCM, hypertrophic cardiomyopathy; LSS, lumbar spinal stenosis; M, male; N, non-endemic area; OH, orthostatic hypotension; Paf, paroxysmal atrial fibrillation; (−), absent; Initial diagnosis was defined as the diagnosis assigned before the definitive diagnosis of ATTRv amyloidosis.

**Table 3:** Longitudinal course, treatment history, and outcomes of individual patients with ATTRv amyloidosis.

Patient No.	Disease Duration (Years)	Longitudinal Manifestations						Treatment	Outcome
		Sensory Disturbances	Muscle Weakness	Autonomic Symptoms	Cardiac Symptoms	Ocular Symptoms	CTS		
1	7	(+)	(+)	(+)	(+)	(−)	(+)	(−)	Dead (cachexia)
2	13	(+)	(−)	(+)	(+)	(+)	(−)	T, P, V	Dead (heart failure)
3	15	(+)	(−)	(+)	(+)	(+)	(+)	T, P, V	Alive
4	5	(+)	(−)	(+)	(+)	(−)	(+)	T	Dead (heart failure)
5	14	(+)	(+)	(+)	(+)	(+)	(+)	P, V	Dead (cerebellar hemorrhage)
6	12	(−)	(−)	(−)	(+)	(+)	(−)	T, P, V	Alive
7	14	(+)	(+)	(+)	(+)	(+)	(−)	T, P, V	Alive
8	12	(+)	(−)	(−)	(+)	(+)	(−)	T, P, V	Alive
9	16	(+)	(+)	(+)	(+)	(+)	(−)	P, V	Dead (heart failure)
10	7	(+)	(−)	(+)	(+)	(+)	(+)	P, V	Alive
11	6	(+)	(−)	(−)	(+)	(−)	(+)	T, V	Alive
12	7	(+)	(−)	(+)	(+)	(−)	(+)	V	Alive
13	8	(+)	(−)	(+)	(−)	(−)	(+)	P, V	Alive
14	12	(+)	(−)	(+)	(−)	(−)	(−)	V	Alive
15	4	(+)	(+)	(+)	(+)	(−)	(−)	V	Alive
16	5	(+)	(+)	(+)	(+)	(−)	(−)	V	Alive
17	1	(+)	(−)	(+)	(+)	(+)	(−)	V	Alive
18	11	(−)	(−)	(+)	(+)	(−)	(−)	V	Alive

Abbreviations: ATTRv, hereditary transthyretin amyloidosis; CTS, carpal tunnel syndrome; P, Patisiran; T, tafamidis; V, vutrisiran; (−), absent; (+), present. The listed manifestations refer to symptoms documented during the longitudinal course of the disease. Disease duration was defined as the interval from disease onset to death or the final follow-up. The final follow-up for the cohort was conducted in December 2025. The clinical manifestations were evaluated by specialists in each department and considered to be associated with ATTRv amyloidosis. No patient received overlapping treatment with different disease-modifying drugs.

## Data Availability

The raw data supporting the conclusions of this article will be made available by the authors on request.

## Supplementary Materials

The following supporting information can be downloaded at: https://www.mdpi.com/article/10.3390/medicina62050907/s1, Figure S1: Change in TTR levels and cardiac parameters after switching from patisiran to vutrisiran. Table S1: TTR variants identified in this cohort. Table S2: Detailed disease-modifying therapy histories of individual patients with ATTRv amyloidosis.

## Abbreviations

The following abbreviations are used in this manuscript:

ATTRv	Hereditary transthyretin amyloidosis (v = variant)
CIDP	Chronic inflammatory demyelinating polyneuropathy
CTS	Carpal tunnel syndrome
DMDs	Disease-modifying drugs
HCM	Hypertrophic cardiomyopathy
IVST	Interventricular septal thickness
LSS	Lumbar spinal stenosis
LVEF	Left ventricular ejection fraction
M	Male
N	Non-endemic area
NA	Not available
NT-proBNP	N-terminal prohormone of brain natriuretic peptide
OH	Orthostatic hypotension
P	Patisiran
Paf	Paroxysmal atrial fibrillation
T	Tafamidis
TTR	Transthyretin
V	Vutrisiran
