# Supplementary material for: Clinical Practice and Diagnostic Trends in Hereditary Transthyretin Amyloidosis: A 25-Year Observational Study
Source: Medicina (Kaunas). 2026 May 7;62(5):907. doi: 10.3390/medicina62050907 (PMC13208621; doi:10.3390/medicina62050907)
Supplement: Supplementary file 1 [file medicina-62-00907-s001.zip › Suppl. Fig. S1_20260504.pdf]

Supplemental Fig. S1

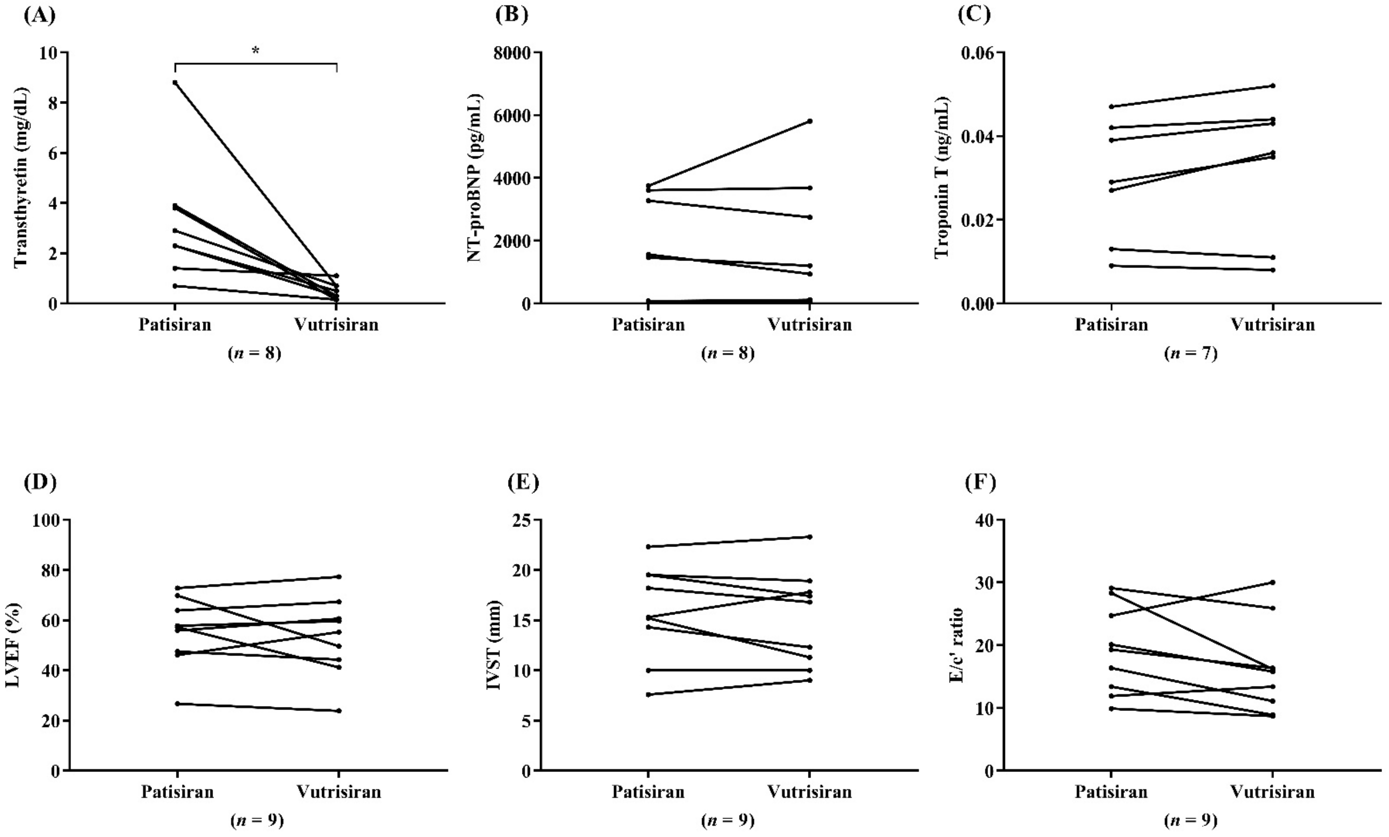

Supplemental Fig. S1. Change in TTR levels and cardiac parameters after switching from patisiran to vutrisiran.

This figure provides the levels of transthyretin (TTR) (A), N-terminal prohormone of brain natriuretic peptide (NT-proBNP) (B), troponin T (C), left ventricular ejection fraction (LVEF) (D), interventricular septal thickness (IVST) (E), and E/e′ ratio (F) 1 year after the switch from patisiran to vutrisiran. *$p < 0.05$.
