# Supplementary material for: Clinical Practice and Diagnostic Trends in Hereditary Transthyretin Amyloidosis: A 25-Year Observational Study
Source: Medicina (Kaunas). 2026 May 7;62(5):907. doi: 10.3390/medicina62050907 (PMC13208621; doi:10.3390/medicina62050907)
Supplement: Supplementary file 1 [file medicina-62-00907-s001.zip › Suppl. Table. S1_20260504.pdf]

Suppl. Table. S1.
TTR variants identified in this cohort.

TTR variant	HGVS protein nomenclature	cDNA change	Pathogenicity classification	Number of patients, n(%)
Val30Met	p.Val50Met	c.148G>A	Pathogenic	10 (55.6)
Tyr114Ser	p.Tyr134Ser	c.401A>C	Pathogenic	4 (22.2)
Glu89Lys	p.Glu109Lys	c.325G>A	Pathogenic	2 (11.1)
His56Arg	p.His76Arg	c.227A>G	Pathogenic	1 (5.6)
Thr49Ile	p.Thr69Ile	c.206C>T	Pathogenic	1 (5.6)

Abbreviations: A, Adenine; Arg, Arginine; C, Cytosine; cDNA, complementary deoxyribonucleic acid; G, Guanine; Glu, Glutamic acid; HGVS, human genome variation society; His, Histidine; Ile, Isoleucine; Lys, Lysine; Met, Methionine; Ser, Serine; T, Thymine; Thr, Threonine; TTR, transthyretin; Tyr, Tyrosine; Val, Valine.
