# Supplementary material for: Clinical Practice and Diagnostic Trends in Hereditary Transthyretin Amyloidosis: A 25-Year Observational Study
Source: Medicina (Kaunas). 2026 May 7;62(5):907. doi: 10.3390/medicina62050907 (PMC13208621; doi:10.3390/medicina62050907)
Supplement: Supplementary file 1 [file medicina-62-00907-s001.zip › Suppl. Table. S2_20260504.pdf]

Suppl. Table. S2.
Detailed disease-modifying therapy histories of individual patients with ATTRv amyloidosis.

Patient no.	Total treatment duration (month)	Treatment start date and duration (month)			Reasons for changing treatment
		Tafamidis	Patisiran	Vutrisiran	
1	0	-	-	-	N/A
2	141	November 2013 (75)	February 2020 (34)	December 2022 (32)	Clinical progression, convenience of treatment administration, and patient preference
3	141	March 2014 (68)	November 2019 (37)	December 2022 (36)	Clinical progression, convenience of treatment administration, and patient preference
4	1	June 2017 (1)	-	-	N/A
5	119	-	May 2015 (93)	February 2023 (26)	Clinical progression, convenience of treatment administration, and patient preference
6	117	March 2016 (45)	December 2019 (36)	December 2022 (36)	Clinical progression, convenience of treatment administration, and patient preference
7	117	March 2016 (63)	June 2021 (19)	January 2023 (35)	Clinical progression, convenience of treatment administration, and patient preference
8	97	November 2017 (33)	August 2020 (29)	January 2023 (35)	Clinical progression, convenience of treatment administration, and patient preference
9	61	-	July 2020 (29)	December 2022 (32)	Convenience of treatment administration, and patient preference
10	56	-	April 2021 (20)	December 2022 (36)	Convenience of treatment administration, and patient preference
11	53	July 2021 (18)	-	January 2023 (35)	Clinical progression and patient preference
12	36	-	-	December 2022 (36)	N/A
13	50	-	October 2021 (19)	May 2023 (31)	Convenience of treatment administration, and patient preference
14	34	-	-	February 2023 (34)	N/A
15	29	-	-	July 2023 (29)	N/A
16	12	-	-	December 2024 (12)	N/A
17	10	-	-	February 2025 (10)	N/A
18	3	-	-	September 2025 (3)	N/A

Abbreviations: ATTRv, hereditary transthyretin amyloidosis; N/A; not applicable.
Total treatment duration was calculated as the sum of the durations of tafamidis, patisiran, and vutrisiran therapy until December 2025 or the date of death, whichever occurred first. No patient received overlapping disease-modifying therapies.
